# The Impact of Inflammatory Bowel Disease Related Content Shared on Twitter: Are We Reaching Other Health Care Professionals or Patients?

**DOI:** 10.1093/crocol/otab053

**Published:** 2021-08-02

**Authors:** Aline Charabaty

**Affiliations:** Department of Gastroenterology and Hepatology, Johns Hopkins School of Medicine, Washington, District of Columbia, USA

Several billion global users have accounts on the 3 main social media platforms (Facebook, Instagram, and Twitter), making online health communities readily accessible to patients. Patients with inflammatory bowel disease (IBD) have been active on social media for several years, seeking medical information and support from other patients, traditionally in closed Facebook groups. In a survey^1^ by Reich et al of 1960 patients with IBD, 1/3 of respondents reported using social media within the week prior to obtain or post IBD-related content. Patients with IBD living in rural areas with limited access to physicians often turn to social media to obtain information and would like to see their gastroenterologist have a social media account with IBD content, with up to 1/3 preferring Facebook as a platform.^2^ In addition, patient advocates have taken on open social media platforms such as blogs, podcasts, Instagram, and Twitter, to advocate for the IBD community needs and to use their informed voice to translate medical information to other patients.

It is only in recent years that GI professional societies and gastroenterologists have taken interest in social media, in particular Twitter, as a tool to amplify medical information from meetings or publications and research work; it has rapidly become a powerful media to quickly and effectively share medical knowledge, connect with peers, and grow one’s professional network.^3^ A recently published study^4^ looking at the demographics of individual gastroenterologists with the most read posts on Twitter, found that IBD specialists from academic institutions ranked highest compared to other subspecialties. Moreover, organized Twitter discussions and journal clubs, such as @MondayNightIBD, @IbdClub, and @GIJournal have been successful in amplifying multidisciplinary scientific exchanges between IBD specialists and different clinicians from academic and private institutions.

The study by Kesavarapu et al published in this issue of *CC360* aim to assess the nature of interaction on Twitter (replies, retweets, and likes) between IBD specialists from different institutions and the type of users (health care professionals, patients, professional societies) who responded to healthcare professional (HCP) tweets, during a period of 2 months in 2019. They found that knowledge sharing, specifically about research, is the most common theme for tweets by HCPs, followed by endorsement by one HCP of another HCP’s professional contributions (eg, lectures, promotions, publications, etc.). Interactions (replies and retweets) are largely driven by HCPs (73%) with a focus on knowledge sharing, in particular disseminating IBD research and related management, while 19% of interactions occur between HCPs and patients/advocates. The authors concluded that HCP–HCP engagement occurs more often than HCP–patients on Twitter, and that Twitter use by HCP for patient education is not yet optimized.

However, these findings of low HCP–patients tweets interactions do not minimize the importance and the impact of HCP sharing IBD content on Twitter to reach and disseminate accurate medical information to patients. Of 267 replies to tweets that were analyzed, 53 came from patients/advocates compared to the 200 replies from HCPs, which is still a substantial interaction, highlighting the opportunity offered by Twitter for HCP and patients to interact with each other. Patient advocates were more likely to engage HCPs with comments, while non-advocate patients used likes and retweets more often. This highlights the fact that non-advocate patients could feel intimidated commenting on HCP posts, and might often be silent observers/learners. Moreover, the authors limited their analysis to the users of the first 25 replies or likes to a tweet, and there is a possibility that HCP could often be the first to interact with a medical tweet rather than patients. With advocates having the potential to report back to other patients, it would be interesting to see the patients’ interactions that follow a retweet by an advocate of a HCP tweet and whether advocates’ input facilitates more patients’ comments and interactions. Finally, results of HCP–patients interaction could be different by IBD content with potentially higher interest for topics such as social support, insurance coverage, diet, mental health, symptoms management, or current hot topics such as IBD and COVID/COVID vaccine. A UK survey^5^ evaluated social media use in patients with IBD during the SARS-CoV2 pandemic: half stated that one-on-one contact with a gastroenterologist via social media was desirable, and 41% wanted a gastroenterologist to answer patients’ questions in a dedicated social media group. Clinicians who wish to disseminate medical information to HCP and patients alike, should consider sharing more “digestible” content and wording in their tweets.

The COVID pandemic has likely boosted the use of social media by HCP and patients alike and promoted its growth as a powerful tool to disseminate IBD medical knowledge for all stakeholders. Results from Kesavarapu et al study and others highlight that Twitter is becoming an effective platform to share medical knowledge and gather medical expertise around an IBD topic. This exchange of medical information between HCP–HCP and HCP–patients not only amplifies the reach of IBD education, but can lead to improved IBD care, as well as effective networking with peers.^6^ For gastroenterologists planning to expand their presence on social media,^7^ the first step is to choose the platform that fits their goals and target audience as well as their personality and the time they plan to dedicate to social media work. Similar to a business card, the profile picture, handle and bio should introduce you and reflect who you are and what your professional interests are. Posts should provide credible information (from conferences, articles, societies guidelines, etc.) that helps close a knowledge gap, in a way that is easy and attractive to read: choose words that make the information digestible to specialists and patients alike, include a link to journal article, a summary image or video, use searchable hashtags # judiciously, and give credit to the authors or organization behind the information with a tag. Make your post interactive by providing an opinion, asking a relevant question related to the information shared, or using a poll, and tag people you wish to engage. Posts can include education beyond medical information (eg, “how to write an editorial,” “how to prepare for an IBD fellowship”) or highlight advocacy work of interest and inspire others (working for better medical coverage for patients, advancing diversity in GI, fighting physician burnout…). Whether the goal is to promote professional and/or patient education, disseminate one’s research and work, increase the reach of one’s practice or institution, or advocate for the advancement of our profession or health care, it is important to maintain a positive, honest, and inclusive presence on social media. Like in any social setting, it is critical to be genuine and respectful in what is said or posted, share regularly high-quality content, and encourage and amplify others’ voice and work. By effectively (and generously) engaging with like-minded people (from gastroenterologists of all stages of career to non-GI clinicians, from GI societies, and GI journals to patients), gastroenterologists can increase their network circle on a national and international level, amplify their professional and advocacy work, and create career and academic opportunities for themselves as well as for others, as mentors and sponsors. Indeed several papers published in peer-reviewed journals were born from discussions and collaborations on Twitter,^6,8^ high impact GI journals have developed social media editorial boards, and scholarly work on social media is now increasingly recognized by academic institutions for promotion and career advancement.^9^

IBD physicians and clinicians presence on social media has revolutionized the way we approach IBD education and communication with peers and patients. In my opinion, this community will continue to embrace, innovate, and optimize its use of social media, all while integrating it to more traditional platforms of professional and patient education, scholarly work and advocacy.
